# Morphology, complete mitochondrial genome, and molecular phylogeny of *Rhabdias macrocephalum* n. sp. (Nematoda: Rhabdiasidae) from *Diploderma splendidum* (Reptilia: Agamidae)[Fn FN1]

**DOI:** 10.1051/parasite/2024046

**Published:** 2024-08-14

**Authors:** Jia-Lu Zeng, Hui-Xia Chen, Hong-Ru Xu, Liang Li

**Affiliations:** 1 Hebei Collaborative Innovation Center for Eco‐Environment, Hebei Key Laboratory of Animal Physiology, Biochemistry and Molecular Biology, College of Life Sciences, Hebei Normal University 050024 Shijiazhuang Hebei Province PR China; 2 Hebei Research Center of the Basic Discipline Cell Biology, Ministry of Education Key Laboratory of Molecular and Cellular Biology 050024 Shijiazhuang Hebei Province PR China

**Keywords:** Zooparasitic nematodes, Rhabdiasidae, Integrative taxonomy, Mitochondrial genome, Phylogeny

## Abstract

Species of the genus *Rhabdias* Stiles & Hassall, 1905 are common parasitic nematodes occurring in the lungs of amphibians and reptiles worldwide. In the present study, *Rhabdias macrocephalum* n. sp. is described using integrated morphological methods (light and scanning electron microscopy) and molecular approaches (sequencing of the nuclear 28S and ITS regions, and mitochondrial *cox1*, *cox2*, and 12S genes) based on specimens collected from the green striped tree dragon *Diploderma splendidum* (Barbour & Dunn) (Reptilia: Agamidae) in China. The complete mitochondrial genome of *R. macrocephalum* n. sp. was sequenced and annotated: it is 14,819 bp in length, including 12 protein coding genes (missing *atp*8), 22 tRNA genes, 2 rRNA genes and three non-coding regions. The gene arrangement of *R. macrocephalum* n. sp. is different from all of the currently available mitogenomes of nematodes and represents a novel type of mitochondrial gene arrangement reported in Nematoda. Molecular phylogenetic results based on the ITS + 28S data support the monophyly of *Entomelas*, *Pneumonema*, *Serpentirhabdias*, and *Rhabdias*, and showed *R. macrocephalum* n. sp. forming a most basal lineage in *Rhabdias*.

## Abstract (fr)

Les espèces du genre *Rhabdias* Stiles & Hassall, 1905 sont des nématodes parasites courants présents dans les poumons des amphibiens et des reptiles du monde entier. Dans cette étude, *Rhabdias macrocephalum* n. sp. est décrit à l’aide de méthodes morphologiques intégrées (microscopie optique et électronique à balayage) et d’approches moléculaires (séquençage des régions nucléaires 28S et ITS et des gènes mitochondriaux *cox1*, *cox2* et *12S*) basées sur des spécimens collectés chez le lézard *Diploderma splendidum* (Barbour & Dunn) (Reptilia : Agamidae) de Chine. Le génome mitochondrial complet de *R. macrocephalum* n. sp. a été séquencé et annoté : il a une longueur de 14 819 pb, dont 12 gènes codants pour des protéines (*atp8* manquant), 22 gènes d’ARNt, 2 gènes d’ARNr et trois régions non codantes. L’arrangement génétique de *R. macrocephalum* n. sp. est différent de tous les mitogénomes de nématodes actuellement disponibles et représente un nouveau type d’arrangement de gènes mitochondriaux signalé chez les nématodes. Les résultats phylogénétiques moléculaires basés sur les données ITS + 28S ont soutenu la monophylie d’*Entomelas*, *Pneumonema*, *Serpentirhabdias* et *Rhabdias*, et ont montré que *R. macrocephalum* n. sp. forme la lignée la plus basale chez *Rhabdias*.

## Introduction

The genus *Rhabdias* (Nematoda: Rhabditida) is the largest group in the family Rhabdiasidae, and currently comprises over 90 nominal species mainly parasitic in the lungs of amphibians and reptiles worldwide [15, 16, 22, 47]. To date, a total of 8 species of *Rhabdias* have been reported in China, namely *R. bicornis* Lu, 1934, *R. incerta* Wilkie, 1930, *R. brevicauda* Chu, 1936, *R. nipponica* Yamaguti, 1935, *R. bufonis* (Schrank, 1788), *R. globocephala* Kung & Wu, 1945, *R. japalurae* Kuzmin, 2003, and *R. kafunata* Sata, Takeuchi & Nakano, 2020 [14, 21, 29, 43, 51, 52]. However, our present knowledge of the species composition of *Rhabdias* nematodes in China is still far from complete.

It is not easy to precisely identify specimens of *Rhabdias* to species level based only on morphological characters, due usually to a lack of males and the extraordinary morphological similarity in females. Recently, some genetic data [i.e., large nuclear ribosomal DNA (28S), internal transcribed spacer (ITS), mitochondrial cytochrome c oxidase subunit 1 (*cox*1), and 12S small subunit ribosomal RNA gene] and mitochondrial genomes have been successfully used to identify species, discover sibling or cryptic species, and evaluate evolutionary relationships of Rhabdiasidae [1, 15, 16, 28, 31, 36, 46, 47, 52]. However, the current genetic database, especially the mitogenomes for the rhabdiasid nematodes, remains very insufficient. To date, only *R. bufonis* and *R. kafunata* have been reported for the complete mitochondrial genomes in the Rhabdiasidae [28, 52].

In the present study, a new species of *Rhabdias* collected from the green striped tree dragon *Diploderma splendidum* (Barbour & Dunn) (Reptilia: Agamidae) in China was precisely identified using integrated morphological methods (light and scanning electron microscopy) and molecular approaches (sequencing of the nuclear 28S and ITS regions and mitochondrial *cox*1, *cox*2, and 12S genes). Additionally, in order to enrich the mitogenomic data and reveal the patterns of mitogenomic evolution of the Rhabdiasidae, the complete mitochondrial genome of this new species was sequenced and annotated. Moreover, in order to determine the phylogenetic position of this new species within *Rhabdias*, phylogenetic analyses were performed based on the 28S + ITS sequences, using maximum likelihood (ML) and Bayesian inference (BI), respectively.

## Materials and methods

### Morphological observation

In 2021, a total of 26 nematode specimens of *Rhabdias* were sent to the author’s (Li L.) laboratory for species identification, which were recovered from the lung of a dead green striped tree dragon *D. splendidum* by a local veterinarian in Qinzhou, Guangxi Zhuang Autonomous Region, China. Specimens were fixed and stored in 80% ethanol until the morphological study. For light microscopy, nematode specimens were cleared in 50% glycerin, then examined and photographed using a Nikon^®^ optical microscope (Nikon ECLIPSE Ni-U, Nikon Corporation, Tokyo, Japan). For scanning electron microscopy (SEM), the anterior and posterior ends of specimens were transferred to 4% formaldehyde solution, then post-fixed in 1% OsO_4_, dehydrated via an ethanol series and acetone and critical point dried. The specimens were coated with gold and examined using a Hitachi S-4800 scanning electron microscope (Hitachi Ltd., Tokyo, Japan) at an accelerating voltage of 20 kV. All measurements in the text are in micrometers unless otherwise stated. Type specimens were deposited in the College of Life Sciences, Hebei Normal University, Hebei Province, and the National Zoological Museum, Beijing, China.

### Molecular procedures

A total of three female specimens were randomly selected for the molecular analysis. Genomic DNA from each individual was extracted using a Column Genomic DNA Isolation Kit (Shanghai Sangon, Shanghai, China), according to the manufacturer’s instructions. DNA was eluted in elution buffer and kept at −20 °C until use. The primers and cycling conditions for amplifying different target regions by polymerase chain reaction (PCR) are provided in Table 1. All PCR reactions were performed in 50 μL consisting of 10 mM Tris HCl at pH 8.4, 50 mM KCl, 3.0 mM MgCl_2_, 250 μM of each dNTP, 50 pmol of each primer, and 1.5 U of Taq polymerase (Takara Bio Inc., Kusatsu, Shiga, Japan) in a thermocycler (model 2720; Applied Biosystems, Thermo Fisher Scientific, Waltham, MA, USA).

**Table 1:** The primers and cycling conditions for amplifying different target regions by polymerase chain reaction (PCR) in the present study*.*

Primer	Sequence 5′-3′	Cycling condition	Source
ITS regions (ITS-1 + 5.8S + ITS-2)	#93: 5′-TTGAACCGGGTAAAAGTCG-3′	94 °C for 3 min	[7]
		94 °C for 30 s	
	#94: 5′-TTAGTTTCTTTTCCTCCGCT-3′	54 °C for 30 s	
		72 °C for 60 s (35 cycles)	
		72 °C for 7 min	
28S	#500: 5′-ACTTTGAAGAGAGAGTTCAAGAG-3′	94 °C for 3 min	[7]
		94 °C for 30 s	
	#501: 5′-TCGGAAGGAACCAGCTACTA-3′	54 °C for 30 s	
		72 °C for 60 s (35 cycles)	
		72 °C for 7 min	
*cox*1	LCO1490: 5′-GGTCAACAAATCATAAAGATATTGG-3′	95 °C for 3 min	[8]
		95 °C for 30 s	
	HCO2198: 5′-TAAACTTCAGGGTGACCAAAAAATCA-3′	50 °C for 30 s	
		72 °C for 90 s (45 cycles)	
		72 °C for 10 min	
12S	12S-F: 5′-GTTCCAGAATAATCGGCTA-3′	94 °C for 3 min	[6]
		94 °C for 45 s	
	12S-R: 5′-ATTGACGGATG(AG)TTTGTACC-3′	48 °C for 45 s	
		72 °C for 1 min (35 cycles)	
		72 °C for 5 min	
*cox*2	*cox*2-F: 5′-AGGTATAAAACTGTGATTTGCACCA-3′	95 °C for 15 min	Present study
		95 °C for 45 s	
	*cox*2-R: 5′-TGTTTTCTGGCAGTTTGTTTTCT-3′	48 °C for 45 s	
		72 °C for 1 min (35 cycles)	
		72 °C for 5 min	

PCR products were checked on GoldView-stained 1.5% agarose gel and purified by the Column PCR Product Purification Kit (Shanghai Sangon). Sequencing for each sample was carried out for both strands using a DyeDeoxyTerminator Cycle Sequencing Kit v.2 (Applied Biosystems). The 28S, ITS, *cox*1, *cox*2, and 12S sequences obtained herein were deposited in the National Center for Biotechnology Information (NCBI) database (http://www.ncbi.nlm.nih.gov).

### Mitochondrial genome sequencing, assembly, and annotation

A total of 30 Gb clean genomic data were generated using the Pair-End 150 sequencing method on the Illumina NovaSeq 6000 platform by Novogene (Tianjin, China). The complete mitochondrial genomes were assembled using GetOrganelle v1.7.2a [12]. Protein coding genes (PCGs), rRNAs, and tRNAs were annotated using MitoS web server (http://mitos2.bioinf.uni-leipzig.de/index.py) and MitoZ v2.4 [33]. The open reading frame (ORF) of each PCG was confirmed manually by the web version of ORF finder (https://www.ncbi.nlm.nih.gov/orffinder/). The “lost” tRNA genes ignored by both MitoS and MitoZ, were identified using BLAST based on a database of the existing tRNA sequences of nematodes. The secondary structures of tRNAs were predicted by ViennaRNA module [9], building on MitoS2 [2] and RNAstructure v6.3 [40], followed by manual correction. MitoZ v2.4 was used to visualize and depict gene element features [33]. The base composition, amino acid usage, and relative synonymous codon usage (RSCU) were calculated by Python script, which refers to Codon Adaptation Index (CAI) [23]. The total length of the base composition included ambiguous bases. The base skew analysis was used to describe the base composition of nucleotide sequences. The complete mitochondrial genome of this new species obtained was deposited in the NCBI database (http://www.ncbi.nlm.nih.gov).

### Phylogenetic analyses

Phylogenetic analyses of rhabdiasid nematodes were performed based on the ITS + 28S sequences using maximum likelihood (ML) with IQ-TREE [34] and Bayesian inference (BI) with MrBayes [41]. *Caenorhabditis elegans* (Rhabditida: Rhabditidae) was chosen as the out-group. The in-group included 46 rhabdiasid species representing six genera. Detailed information on species included in the phylogenetic analyses is provided in Table 2. Genes were aligned separately using the MAFFT v7.313 multiple sequence alignment program under the iterative refinement method of E-INS-I [13]. In addition, partially ambiguous bases were manually inspected and removed. The aligned and pruned sequences were concatenated into a matrix by PhyloSuite v1.2.2. The TVM + F+I + I+R2 model was selected for ML analyses. The GTR + F+G4 models were selected for BI analyses. Reliabilities for ML inference were tested using 1000 bootstrap replications, and BIC analysis was run for 5 × 10^6^ MCMC generations.

**Table 2:** Detailed information on the representatives of Rhabdiasidae with their genetic data included in the phylogenetic analyses.

Species	Host	Locality	GenBank ID for ITS region	GenBank ID for 28S region	References
**Ingroup**					
** Rhabdiasidae**					
** *Rhabdias***					
* R. africanus*	*Sclerophrys gutturalis* (Amphibia: Bufonidae)	Sub-Saharan Africa	MG428407	MG428407	[16]
* R. cf. africanus*	*Hylarana galamensis* (Amphibia: Ranidae)	Nigeria	KF999598	KF999598	[50]
* R. ambystomae*	*Ambystoma maculatum* (Amphibia: Ambystomatidae)	USA	KF999590	KF999590	[50]
* R. americanus*	*Anaxyrus americanus* (Amphibia: Bufonidae)	USA	KF999589	KF999589	[50]
* R. bakeri*	*Lithobates sylvatica* (Amphibia: Ranidae)	USA	DQ264770	DQ264770	[49]
* R. bufonis*	*Rana temporaria* (Amphibia: Ranidae)	Ukraine	KF999593	KF999593	[50]
* R. cf. bufonis*	*Bombina bombina* (Amphibia: Discoglossidae)	Ukraine	KF999606	KF999606	[50]
* R. bulbicauda*	*Bufo* sp. (Amphibia: Bufonidae)	Nepal	KF999600	KF999600	[50]
* R. bermani*	*Salamandrella keyserlingii* (Amphibia: Hynobiidae)	Russia	KF999610	KF999610	[50]
* R. breviensis*	*Leptodactylus fuscus* (Amphibia: Leptodactylidae)	Brazil	MH516070	MH516106	[38]
* R. delangei*	*Strongylopus grayii* (Amphibia: Pyxicephalidae)	South Africa	MT298095	MT298095	[17]
* R. elegans*	*Bufo* sp. (Amphibia: Bufonidae)	Argentina	KF999604	KF999604	[50]
* R. engelbrechti*	*Phrynomantis bifasciatus* (Amphibia: Microhylidae)	South Africa	MG428406	MG428406	[16]
* R. fuelleborni*	*Rhinella diptycha* (Amphibia: Bufonidae)	Brazil	OP651065	OP651188	[39]
* R. guaianensis*	*Leptodactylus podicipinus* (Amphibia: Leptodactylidae)	Brazil	OP972545	OP972542	[1]
* R. joaquinensis*	*Lithobates blairi* (Amphibia: Ranidae)	USA	KF999594	KF999594	[50]
* R. cf. joaquinensis*	*Lithobates clamitans* (Amphibia: Ranidae)	USA	KF999608	KF999608	[50]
* R. kongmonthaensis*	*Polypedates leucomystax* (Amphibia: Rhacophoridae)	Thailand	KF999599	KF999599	[50]
* R. matogrosensis*	*Leptodactylus macrosternum* (Amphibia: Leptodactylidae)	Brazil	OP972546	OP972541	[1]
* R. nipponica*	*Rana japonica* (Amphibia: Ranidae)	Japan	AB818379	LC671705	[10, 31]
* R. nicaraguensis*	*Norops* sp. (Reptilia: Iguanidae)	Costa Rica	KF999605	KF999605	[50]
* R. pseudosphaerocephala*	*Rhinella schneideri* (Amphibia: Bufonidae)	Brazil	MH516078	MH516078	[38]
* R. picardiae*	*Amietia delalandii* (Amphibia: Pyxicephalidae)	South Africa	MG195567	MG195567	[45]
* R. ranae*	*Rana pipiens* (Amphibia: Ranidae)	USA	DQ264766	DQ264766	[49]
* R. rubrovenosa*	*Bufotes viridis* (Amphibia: Bufonidae)	Ukraine	KF999596	KF999596	[50]
* R. sphaerocephala*	*Bufo bufo* (Amphibia: Bufonidae)	Ukraine	DQ845739	DQ845739	[18]
* R. sylvestris*	*Breviceps sylvestris* (Amphibia: Brevicipitidae)	South Africa	KJ018777	KJ01877	[47]
* R. tarichae*	*Taricha granulosa* (Amphibia: Salamandridae)	USA	OL652879	OL652879	Unpublished
* R. kafunata*	*Bufo gargarizans* (Amphibia: Bufonidae)	China	OR682645	OR682285	[52]
* R. bufonis*	*Bufo gargarizans* (Amphibia: Bufonidae)	China	OR690331	OR690325	[52]
* R. macrocephalum* n. sp.	*Diploderma splendidum* (Reptilia: Agamidae)	China	PP544389	PP544391	Present study
** *Entomelas***					
* E. entomelas*	*Anguis fragilis* (Reptilia: Anguidae)	Ukraine	KF999592	KF999592	[50]
* E. kazakhstanica*	*Pseudopus apodus* (Reptilia: Anguidae)	Ukraine	KF999597	KF999597	[50]
* E. ophisauri*	*Pseudopus apodus* (Reptilia: Anguidae)	Ukraine	KF999595	KF999595	[50]
* E. dujardini*	*Anguis fragilis* (Reptilia: Anguidae)	Ukraine	KF999591	KF999591	[50]
** *Pneumonema***					
* P. tiliquae*	*Tiliqua scincoides* (Reptilia: Scincidae)	Australia	KF999611	KF999611	[50]
* Pneumonema* sp. 1	*Tiliqua scincoides* (Reptilia: Scincidae)	Australia	KF999603	KF999603	[50]
* Pneumonema* sp. 2	*Cyclodomorphus gerrardii* (Reptilia: Scincidae)	Australia	KF999612	KF999612	[50]
** *Serpentirhabdias***					
* S. fuscovenosa*	*Natrix natrix* (Reptilia: Colubridae)	Ukraine	KF999588	KF999588	[50]
* S. cf. fuscovenosa*	*Nerodia erythrogaster* (Reptilia: Colubridae)	USA	MH283885	KF999613	[30, 50]
* S. elaphe*	*Zamenis longissimus* (Reptilia: Colubridae)	Ukraine	MH283884	KF999614	[30, 50]
* S. viperidicus*	*Bothrops moojeni* (Reptilia: Colubridae)	Brazil	MH516095	KX354358	[35, 38]
* S. moi*	*Chironius exoletus* (Reptilia: Colubridae)	Brazil	MH283886	MH283886	[30]
* S. mussuranae*	*Clelia clelia* (Reptilia: Colubridae)	Brazil	MK680941	MK680941	[20]
** *Neoentomelas***					
* N. asatoi*	*Ateuchosaurus pellopleurus* (Reptilia: Scincidae)	Japan	LC631539	LC631539	[42]
** *Kurilonema***					
* K. markovi*	*Plestiodon* spp. (Reptilia: Scincidae)	Japan	LC631542	LC631542	[42]
**Outgroup**					
* Caenorhabditis elegans*	*Megophrys montana* (Amphibia: Pelobatidae)	San Diego, USA	FJ589007	EF417141	[11, 44]

## Results

### Description of *Rhabdias macrocephalum* n. sp. (Figs. 1–3)

urn:lsid:zoobank.org:act:BB1898CA-CD19-4568-9A43-84E2AD6FF185

**Figure 1:**
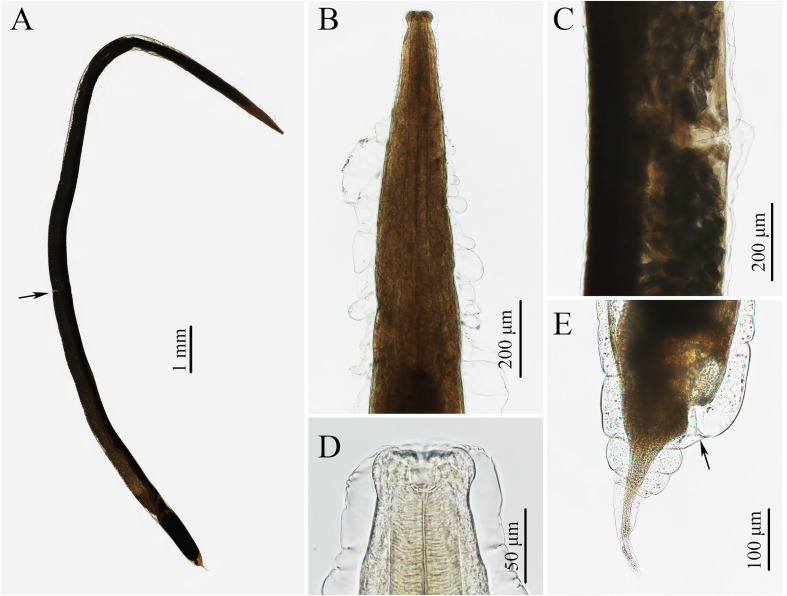
Photomicrographs of *Rhabdias macrocephalum* n. sp. from *Diploderma splendidum* in China. **A:** entire body (vulva arrowed), lateral view; **B:** anterior part of body, lateral view; **C:** region of vulva, lateral view; **D:** cephalic extremity, lateral view; **E:** posterior part of body (cloaca arrowed), lateral view.

*Type host*: Green striped tree dragon *Diploderma splendidum* (Barbour & Dunn) (Reptilia: Agamidae).

*Type locality*: Qinzhou City, Guangxi Zhuang Autonomous Region, China.

*Site in host*: Lung.

*Type specimens*: Holotype: 1 female (HBNU–N–R20240315ZL); paratypes: 22 females (HBNU–N–R20240316ZL), deposited in the College of Life Sciences, Hebei Normal University, Hebei Province; 3 females (NZMC–PN_144–146), deposited in the National Zoological Museum, Beijing, China.

*Etymology*: The specific name refers to the inflated cephalic end of the present specimens.

*GenBank accession:* PP544391–PP544393 (28S), PP544389–PP544390 (ITS), PP533065–PP533067 (*cox*1), PP544387–PP544388 (12S), PP550091 (*cox*2), PP874272 (mitogenome).

**Figure 2:**
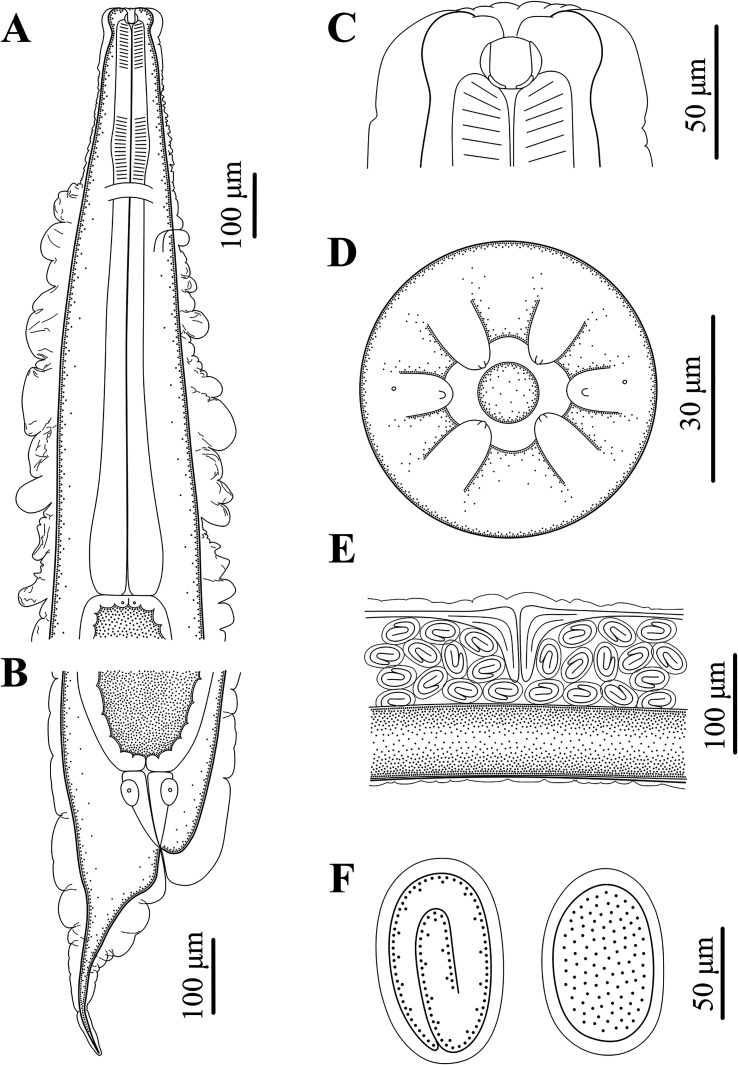
Line drawings of *Rhabdias macrocephalum* n. sp. from *Diploderma splendidum* in China. **A:** anterior part of body, lateral view; **B:** posterior part of body, lateral view; **C:** cephalic extremity, lateral view; **D:** cephalic extremity, apical view; **E:** region of vulva, lateral view; **F:** eggs.

**Figure 3:**
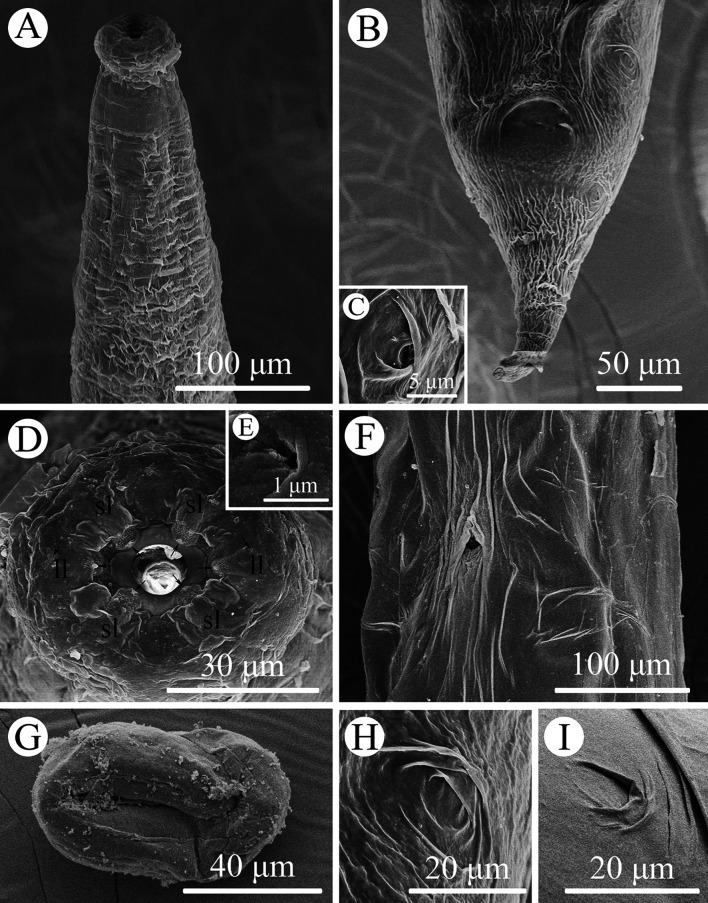
Scanning electron micrographs of *Rhabdias macrocephalum* n. sp. from *Diploderma splendidum* in China. **A:** anterior part of body, lateral view; **B:** tail, ventral view; **C:** magnified image of lateral cuticular pore; **D:** cephalic extremity (single papilla on each lip arrowed), apical view; **E:** magnified image of amphid; **F:** mid-body at level of vulva, sublateral view; **G:** egg with developed larva; **H:** magnified image of lateral cuticular pores on the tail; **I:** magnified image of lateral cuticular pores on the middle of body. *Abbreviations*: sl, submedian lip; ll, lateral lip.

Diagnosis: Body relatively large, gradually tapering from mid-region towards anterior and posterior ends (Fig. 1A). Cephalic extremity conspicuously inflated to form cephalic bulb (Figs. 1B, D, 2A, C, 3A). Cuticle slightly or inconspicuously inflated at anterior region of body, then distinctly inflated to form irregular folds from more or less posterior region of nerve ring (Figs. 1B, 2A), and conspicuously inflated at vulval and caudal region (Figs. 1C, E, 2B, E). Esophagus club-shaped, possessing an indistinct dilation at anterior region of nerve ring, posterior end distinctly expanded to esophageal bulb (Figs. 1B, 2A). Excretory pore just posterior to nerve ring (Figs. 2A, 3E). Tail conical, sharply pointed, abruptly tapering from anus posteriorly, gradually tapering from approximately 1/2 of tail (Figs. 1A, E, 2B, 3B).

General (Based on 10 gravid individuals): Body 14.0–18.0 (17.0) mm long, maximum width 976–1293 (1112). Cuticular pores arranged laterally into 2 longitudinal rows along entire body (Fig. 3B, H, I). Oral opening simple, nearly rounded, surrounded by six small lips (two lateral and four submedian) reduced to elongated elevations (Figs. 2D, 3D); submedian lips located closer to edge of oral opening than lateral lips, each lip bearing single papilla (Figs. 2D, 3D). Small amphids located at base of lateral lip (Figs. 2D, 3E). Vestibulum narrow, cylindrical, cuticularized. Buccal capsule small, cup-like, with well sclerotized walls, 20.0–22.5 (21.5) deep, 25.0–30.0 (26.8) wide (Figs. 1D, 2A, C). Esophagus 870–980 (937) in total length, representing 5.35–6.00 (5.67) % of body length. Nerve ring 251–319 (278) from cephalic extremity. Uteri didelphic and amphidelphic, typical of *Rhabdias*; vulval opening with slightly protruding lips, 8.29–9.76 (9.35) mm from cephalic extremity, representing 53.6–58.5 (56.6) % of body length (Figs. 1A, C, 2E, 3F). Uteri thin-walled, filled with well developed, embryonated or unembryonated eggs (Figs. 1C, 2E, F, 3G). Eggs oval, with smooth thin-shell, 72–116 (91) × 34–68 (49) (*n* = 20). Tail 261–328 (302) long, representing 1.52–2.02 (1.83) % body length.

### Genetic characterization

Three partial 28S sequences of *R. macrocephalum* n. sp. obtained here are all 551 bp, with no nucleotide divergence detected. Pairwise comparison of the partial 28S sequences of *R. macrocephalum* n. sp. obtained here with that of *Rhabdias* available in GenBank, displayed 1.45% (*R. pseudosphaerocephala*, MH516124; *R. breviensis*, MH516101) to 3.58% (*R. tarichae*, MH023521) nucleotide divergence. Two partial ITS sequences of *R. macrocephalum* n. sp. obtained here are both 700 bp, with no nucleotide divergence detected. Pairwise comparison of the partial ITS sequences of *R. macrocephalum* n. sp. obtained here with that of *Rhabdias* spp. available in GenBank, displayed 7.80% (*R. breviensis*, MH516064) to 16.8% (*R. stomatica*, MW522544) nucleotide divergence. Three partial *cox*1 sequences of *R. macrocephalum* n. sp. obtained here are all 655 bp, with no nucleotide divergence detected. Pairwise comparison of the partial *cox*1 sequences of *R. macrocephalum* n. sp. obtained here with that of *Rhabdias* spp. available in GenBank, displayed 8.70% (*R. nipponica*, LC671281) to 15.4% (*R. lamothei*, KC130747) nucleotide divergence. Two partial 12S sequences of *R. macrocephalum* n. sp. obtained here are both 474 bp, with no nucleotide divergence detected. Pairwise comparison of the partial 12S sequences of *R. macrocephalum* n. sp. obtained here with that of *Rhabdias* spp. available in GenBank, displayed 8.91% (*R. engelbrechti*, MG428408) to 11.5% (*R. mariauxi*, FN395318) nucleotide divergence. One partial *cox2* sequence of *R. macrocephalum* n. sp. obtained here is 554 bp. Pairwise comparison of the partial *cox2* sequence of *R. macrocephalum* n. sp. obtained here with that of *Rhabdias* spp. available in GenBank, displayed 11.2% (*R. bufonis*) to 13.2% (*R. kafunata*) nucleotide divergence.

### Characterization of complete mitogenome

**Figure 4:**
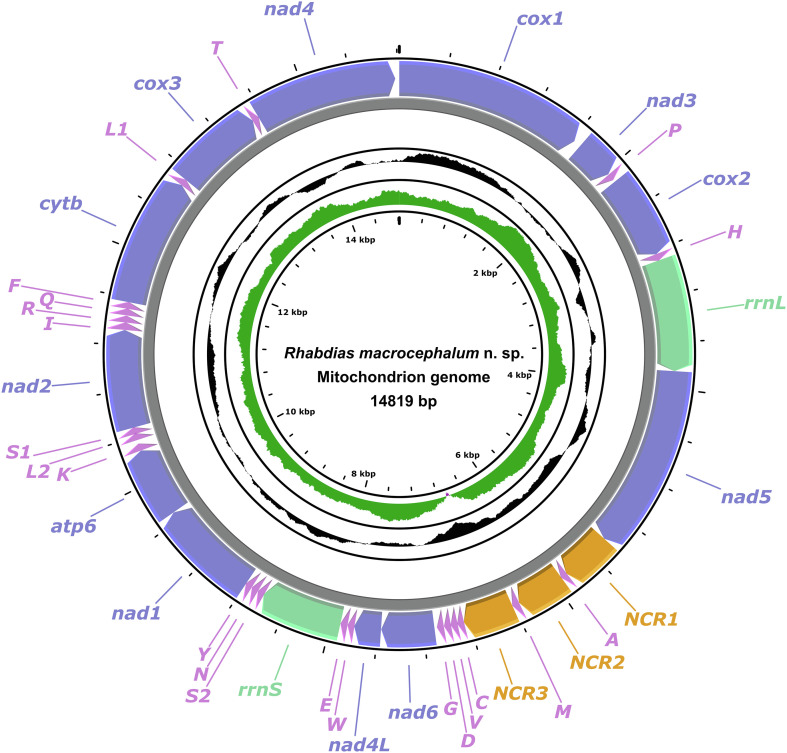
Gene maps of the mitochondrial genomes of *Rhabdias macrocephalum* n. sp. *Abbreviations*: NCR, non-coding region; PCG, protein coding gene; rRNA, ribosomal RNA; tRNA, transfer RNA.

The mitogenome of *R. macrocephalum* n. sp. had 14,819 bp, containing 36 genes, including 12 PCGs (missing *atp*8) (*cox*1–3, *cyt*b, *nad*1–6, *nad*4L and *atp*6), 22 tRNA genes, and 2 rRNA genes (*rrn*L and *rrn*S) (Fig. 4, Table 3). All genes were transcribed from the same DNA strand. There were three non-coding regions in the mitogenome of *R. macrocephalum* n. sp. (NCR1 is 441 bp, between *nad*5 and *tRNA-Ala*; NCR2 is 421 bp, between *tRNA-Ala* and *tRNA-Met*; NCR3 is 439 bp, between *tRNA-Met* and *tRNA-Cys*) (Fig. 4). The nucleotide contents of mitogenome of *R. macrocephalum* n. sp. are provided in Table 4. The overall A + T contents in the mitogenome of *R. macrocephalum* n. sp. was 77.5%, showing a strong nucleotide compositional bias toward A + T (Table 4).

**Table 3:** Annotations and gene organization of *Rhabdias macrocephalum* n. sp. Positive number in the “Gap or overlap” column indicates the length of intergenic sequence, and the negative number indicates the length (absolute number) that adjacent genes overlap (negative sign). The forward strand is marked as “+” and the reverse strand as “−”.

Gene	Type	Start	End	Length	Start Codon	Stop Codon	Anticodon	Strand	Gap or overlap
*cox*1	CDS	1	1623	1623	TTG	TAA		+	64
*nad*3	CDS	1688	2029	342	ATT	TAG		+	6
tRNA-Pro (P)	tRNA	2036	2092	57			UGG	+	21
*cox*2	CDS	2114	2830	717	ATT	TAA		+	1
tRNA-His (H)	tRNA	2832	2887	56			GUG	+	0
*rrn*L	rRNA	2888	3850	963				+	0
*nad*5	CDS	3851	5434	1584	ATT	TAA		+	0
NCR1	Non-coding region	5435	5875	441				+	0
tRNA-Ala (A)	tRNA	5876	5930	55			UGC	+	0
NCR2	Non-coding region	5931	6351	421				+	0
tRNA-Met (M)	tRNA	6352	6414	63			CAU	+	0
NCR3	Non-coding region	6415	6853	439				+	0
tRNA-Cys (C)	tRNA	6854	6910	57			GCA	+	0
tRNA-Val (V)	tRNA	6911	6967	57			UAC	+	1
tRNA-Asp (D)	tRNA	6969	7024	56			GUC	+	6
tRNA-Gly (G)	tRNA	7031	7086	56			UCC	+	23
*nad*6	CDS	7110	7565	456	ATG	TAA		+	2
*nad*4L	CDS	7568	7798	231	ATA	TAG		+	0
tRNA-Trp (W)	tRNA	7799	7854	56			UCA	+	2
tRNA-Glu (E)	tRNA	7857	7918	62			UUC	+	0
*rrn*S	rRNA	7919	8623	705				+	0
tRNA-Ser2 (S2)	tRNA	8624	8677	54			UGA	+	5
tRNA-Asn (N)	tRNA	8683	8738	56			GUU	+	5
tRNA-Tyr (Y)	tRNA	8744	8800	57			GUA	+	0
*nad*1	CDS	8801	9673	873	TTG	TAA		+	1
*atp*6	CDS	9675	10274	600	ATT	TAA		+	0
tRNA-Lys (K)	tRNA	10275	10337	63			UUU	+	34
tRNA-Leu2 (L2)	tRNA	10372	10427	56			UAA	+	0
tRNA-Ser1 (S1)	tRNA	10428	10480	53			UCU	+	0
*nad*2	CDS	10481	11323	843	TTG	TAA		+	0
tRNA-Ile (I)	tRNA	11324	11389	66			GAU	+	0
tRNA-Arg (R)	tRNA	11390	11445	56			ACG	+	6
tRNA-Gln (Q)	tRNA	11452	11506	55			UUG	+	2
tRNA-Phe (F)	tRNA	11509	11563	55			GAA	+	0
*cyt*b	CDS	11564	12673	1110	TTG	TAA		+	1
tRNA-Leu1 (L1)	tRNA	12675	12730	56			UAG	+	0
*cox*3	CDS	12731	13498	768	TTG	TAG		+	1
tRNA-Thr (T)	tRNA	13500	13555	56			UGU	+	0
*nad*4	CDS	13556	14785	1230	TTG	TAG		+	

**Table 4:** Base composition and skewness of *Rhabdias macrocephalum* n. sp.

Location/Species	Total (bp)	A (%)	T (%)	C (%)	G (%)	A + T (%)	AT skew	GC skew
Mitochondrial genome	14,819	29.1	48.4	7.00	15.5	77.5	−0.25	0.38
Protein coding genes (PCGs)	10,377	26.3	50.5	7.15	16.1	76.8	−0.31	0.38
Codon position								
1st codon	3459	30.0	41.8	7.05	21.2	71.8	−0.16	0.50
2nd codon	3459	19.9	51.3	12.4	16.5	71.1	−0.44	0.14
3rd codon	3459	29.1	58.3	2.02	10.5	87.5	−0.33	0.68
tRNAs	1258	34.9	40.9	7.87	16.4	75.8	−0.08	0.35
rRNAs	1668	35.1	41.6	7.67	15.6	76.7	−0.08	0.34
*rrn*L	963	34.2	45.4	6.23	14.2	79.5	−0.14	0.39
*rrn*S	705	36.5	36.5	9.65	17.5	72.9	0.00	0.29
Non-coding region 1	441	32.9	48.1	5.0	14.1	81.0	−0.19	0.48
Non-coding region 2	421	34.7	47.0	5.0	13.3	81.7	−0.15	0.45
Non-coding region 3	439	44.9	46.2	4.8	4.1	91.1	−0.01	−0.08

**Figure 5:**
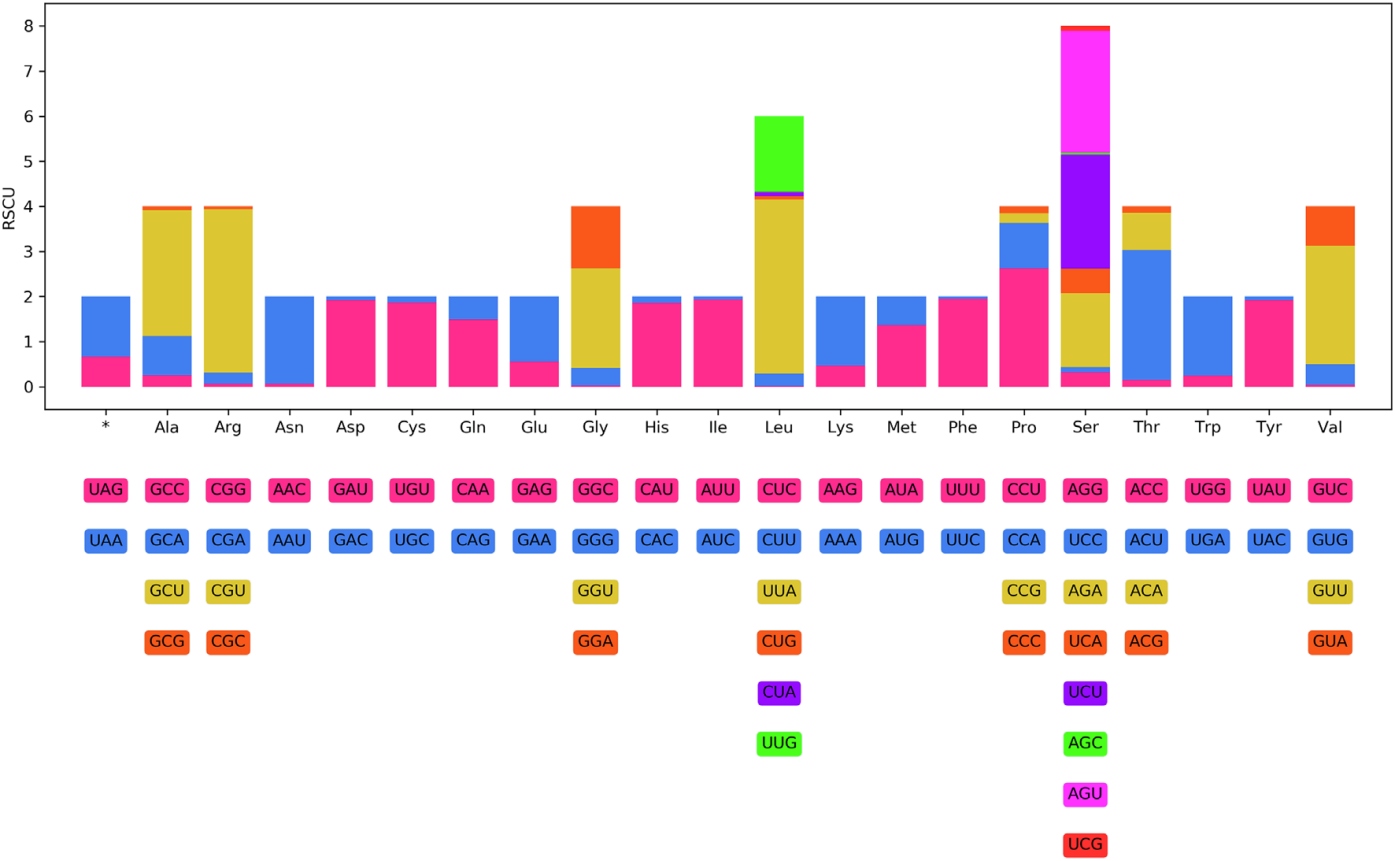
Relative synonymous codon usage (RSCU) of *Rhabdias macrocephalum* n. sp. Codon families (in alphabetical order, from left to right) are provided below the horizontal axis. Values at the top of each bar represent amino acid usage in percentage.

The 12 PCGs of the mitogenome of *R. macrocephalum* n. sp. had 10,377 bp (excluding termination codons), and ranged in size from 231 bp (*nad4L*) to 1623 bp (*cox1*), which encoded 3448 amino acids. Among the 12 PCGs of *R. macrocephalum* n. sp., six genes (*cox*1, *nad*1, *nad*2, *cyt*b, *cox*3, and *nad*4) used TTG as the start codon, followed by ATT for five genes (*nad*3, *cox*2, *nad*5, *nad*4L, and *atp*6), and ATG was used by *nad*6. TAA was the most commonly used termination codon (*cox*1, *cox*2, *nad*5, *nad*6, *nad*1, *atp*6, *nad*2, and *cyt*b), and four genes including *nad*3, *nad*4L, *cox*3, and *nad*4 used TAG (Table 3). The component and usages of codons in the mitogenome of *R. macrocephalum* n. sp. are shown in Figure 5. The lengths of 22 tRNAs of *R. macrocephalum* n. sp. are provided (Table 3).

**Figure 6:**
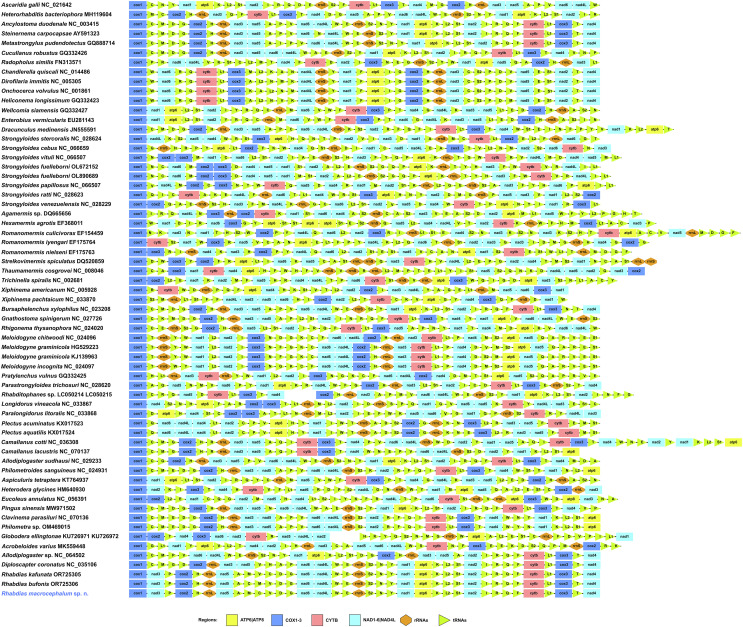
Linearized representation of the nematode mitochondrial gene arrangement of nematodes. The non-coding regions are not indicated.

The 36 gene arrangement in the mitogenomes of *R. macrocephalum* n. sp. differs from any of the arrangement types reported so far for Nematoda. The arrangement in *R. macrocephalum* n. sp. is in the following order: *cox*1, *nad*3, *tRNA-Pro*, *cox*2, *tRNA-His*, *rrn*L, *nad*5, *tRNA-Ala*, *tRNA-Met*, *tRNA-Cys*, *tRNA-Val*, *tRNA-Asp*, *tRNA-Gly*, *nad*6, *nad*4L, *tRNA-Trp*, *tRNA-Glu*, *rrn*S, *tRNA-Ser*2, *tRNA-Asn*, *tRNA-Tyr*, *nad*1, *atp*6, *tRNA-Lys*, *tRNA-Leu*2, *tRNA-Ser*1, *nad*2, *tRNA-Ile*, *tRNA-Arg*, *tRNA-Gln*, *tRNA-Phe*, *cyt*b, *tRNA-Leu*1, *cox*3, *tRNA-Thr*, *nad*4 (Fig. 6).

### Molecular phylogeny of Rhabdiasidae

**Figure 7:**
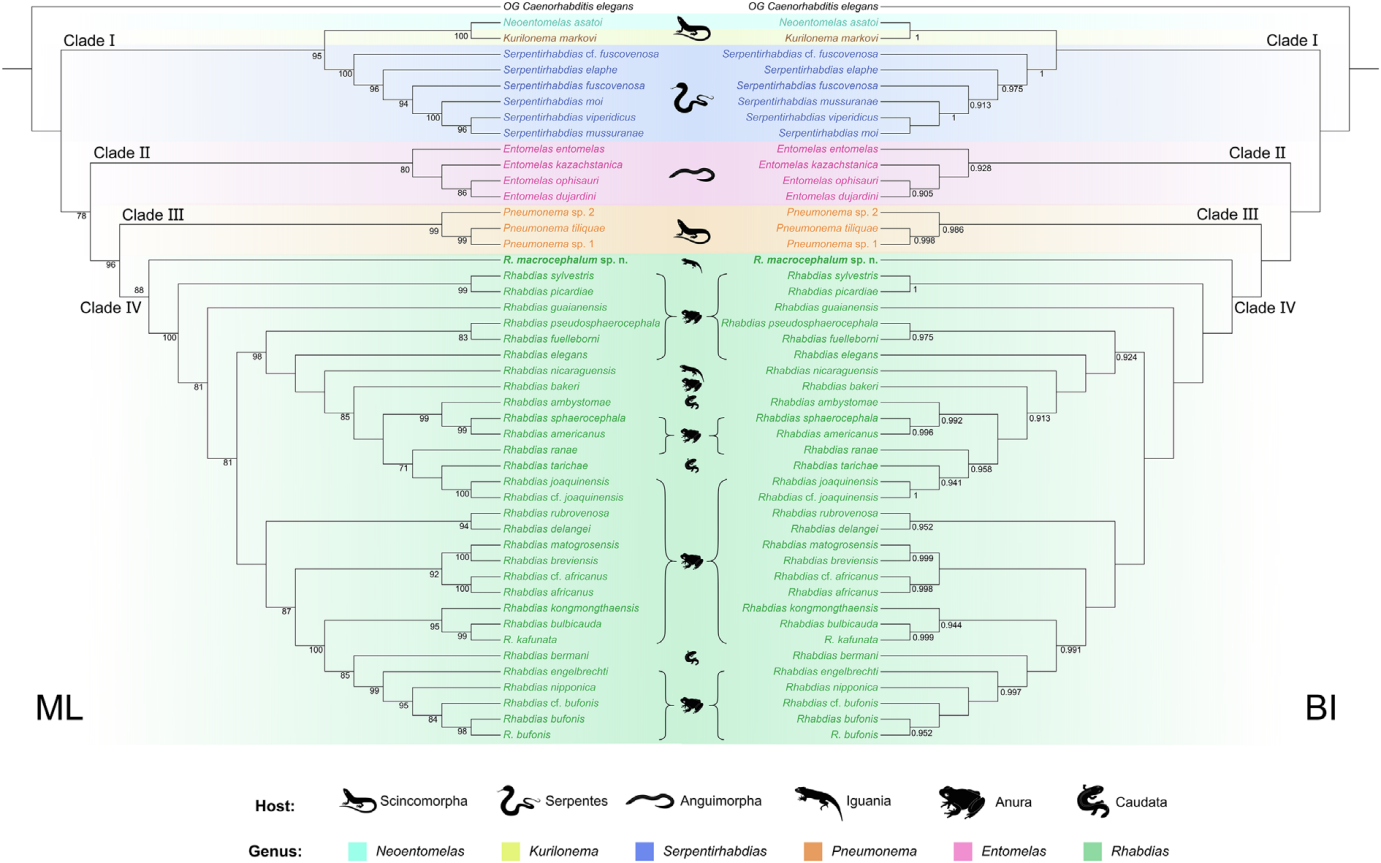
Maximum likelihood (ML) inference and Bayesian inference (BI) based on the ITS + 28S sequence data showing the phylogenetic relationships of representatives of Rhabdiasidae. *Caenorhabditis elegans* Dougherty (Rhabditida: Rhabditidae) was chosen as the out-group. Bootstrap values ≥70 and Bayesian posterior probabilities values ≥0.90 are shown in the phylogenetic trees. Bold indicates *Rhabdias macrocephalum* n. sp.

Phylogenetic results based on the ITS + 28S sequence data using ML and BI methods are almost identical (Fig. 7). The representatives of Rhabdiasidae were divided into four large monophyletic clades (Clade I, II, III, and IV). Clade I comprises species of *Neoentomelas*, *Kurilonema*, and *Serpentirhabdias*. Among them, *Neoentomelas* and *Kurilonema* have a closer relationship than *Serpentirhabdias*. Clade II includes representatives of *Entomelas*. Clade III contains species of *Pneumonema*, which showed a sister relationship with Clade IV, representing *Rhabdias*. In the genus *Rhabdias*, *R. macrocephalum* n. sp. formed a most basal lineage (Fig. 7).

## Discussion

In the genus *Rhabdias*, a total of 21 species have been reported from lizards worldwide [5, 19, 37, 48]. Among them, only four species of *Rhabdias* were recorded from the lizards of the family Agamidae, including *R. japalurae* Kuzmin, 2003, *R. singaporensis* Bursey, Hoong & Goldberg, 2012, *R. mcguirei* Tkach, Kuzmin & Brown, 2011, and *R. odilebaini* Kuzmin, Tkach & Bush, 2012 [5, 19, 21, 48]. *Rhabdias macrocephalum* n. sp. can be easily distinguished from *R. singaporensis* by having a much longer esophagus (0.87–0.98 mm long, representing 5.35–6.00% of body length in *R. macrocephalum vs* 0.497–0.689 mm long, representing approximately 4.00% of body length in the latter) and different location of the excretory pore (just posterior to the nerve ring in the new species *vs* at the level of esophageal-intestinal junction in *R. singaporensis*) [5]. The new species is also different from *R. odilebaini* by having a particular pattern of cuticular inflation (cuticular inflation very narrow or inconspicuous in the anteriormost part and distinctly widening posteriorly from the level of nerve ring or mid-length of esophagus in the new species *vs* cuticle distinctly inflated to form a vesicle swollen in the anteriormost part of the body) and distinctly shorter tail (0.26–0.33 mm long, representing 1.52–2.02% of body length in the new species *vs* 0.36–0.50 mm long, representing 3.10–3.50% of body length in *R. odilebaini*) [19].

With the particular pattern of cuticular inflation, *R. macrocephalum* n. sp. is very similar to *R. japalurae* reported from *Diploderma polygonatum* Hallowell and *D. swinhonis* (Gunther) in Japan (Okinawa Island) and China (Taiwan Island), and *R. mcguirei* reported from *Draco spilopterus* (Wiegmann) in the Philippines [21, 48]. However, *R. macrocephalum* n. sp. can be differentiated from *R. japalurae* by having a distinctly shorter esophagus (0.87–0.98 mm long, representing 5.35–6.00% of body length in the new species *vs* 0.92–1.04 mm long, representing approximately 8.90–9.40% of body length in *R. japalurae*) [21]. The new species also differs from *R. mcguirei* by having a relatively shorter esophagus (esophageal length representing 5.35–6.00% of body length in the new species *vs* esophageal length representing 7.40–14.1% of body length in *R. mcguirei*) and different morphology of the tail (tail with distinct cuticular inflation and abruptly tapering from approximately 1/2 of region *vs* a tail with very narrow or inconspicuous cuticular inflation and abruptly tapering from anterior 1/3 of the region) [48]. Moravec [37] described *R. lacerate* Moravec, 2010 from the common lizard *Lacerta vivipara* Jacquin (Squamata: Lacertidae) in north-western Slovakia. This species with a very small body length (only 1.22–1.34 mm) and unique morphology of the tail tip (possessing 3 small cuticular spikes), is different from *R. macrocephalum* n. sp. Moreover, the other *Rhabdias* spp. reported from lizards are all collected from chameleonid and polychrotid hosts and distributed in tropical Africa, Madagascar, and Central America [3, 4, 19, 24–27, 32]. Additionally, *R. macrocephalum* n. sp. differs from all of these 21 *Rhabdias* spp. reported from lizards, including the four species parasitic in agamids, by having a conspicuously inflated cephalic extremity.

Molecular analyses of the partial 28S, ITS, *cox*1, and 12S sequences of *R. macrocephalum* n. sp. displayed no nucleotide divergence among different individuals, but showed a high level of genetic divergence between this new species and other *Rhabdias* spp. in these genetic makers, which also supports the hypothesis that the present material represents a new species of *Rhabdias*. *Rhabdias macrocephalum* n. sp. represents the ninth species of *Rhabdias* reported in China.

The current mitogenomic database for rhabdiasid nematodes remains very limited. Recently, the complete mitogenomes of *R. kafunata* and *R. bufonis* have been sequenced [52], which represented the only two rhabdiasid species with the mitogenomic data reported. The composition of the mitogenome of *R. macrocephalum* n. sp. [including 12 PCGs (missing *atp*8), 22 tRNA genes, and 2 rRNA genes] is identical to that of *R. kafunata* and *R. bufonis*, but the size of the complete mitogenome of *R. macrocephalum* n. sp. (14,819 bp) is slightly smaller than that of *R. kafunata* (15,437 bp) and *R. bufonis* (15,128 bp). Moreover, there are only three non-coding regions in the mitogenome of *R. macrocephalum* n. sp., but *R. kafunata* and *R. bufonis* have six and four non-coding regions in their mitogenomes, respectively. The mitogenomes of *R. macrocephalum* n. sp., *R. kafunata*, and *R. bufonis* all displayed a strong nucleotide compositional bias toward A + T (75.8–77.5%). To date, there have been 62 types of gene arrangements reported for the mitogenomes of nematodes [52]. The mitogenome of *R. macrocephalum* n. sp. showed a high level of gene rearrangement, which is different from that of *R. kafunata*, *R. bufonis*, and all of other mitogenomes of nematodes available so far, and represented a novel type of gene arrangement reported in Nematoda.

Recently, Zeng et al. [52] provided a basic molecular phylogenetic framework for the Rhabdiasidae based on ITS + 28S sequence data, and determined the systematic position of the Rhabdiasidae in the order Rhabditida using mitogenomic phylogeny. The present phylogenetic results agreed well with this study [52] and also supported the monophyly of *Entomelas*, *Pneumonema*, *Serpentirhabdias*, and *Rhabdias*. It is interesting that the present phylogenetic results displayed *R. macrocephalum* n. sp. forming a most basal lineage in the genus *Rhabdias*, being a sister to all other *Rhabdias* species. In the present phylogeny, only *R. nicaraguensis* Bursey, Goldberg & Vitt, 2007 was collected from a lizard host [4]; however, this species nested in these *Rhabdias* species collected from amphibians in South and North America, and did not display a close affinity with the new species. Additionally, *R. macrocephalum* n. sp. showed a distant relationship to the Eurasian *Rhabdias* species (i.e., *R. bufonis*, *R. kafunata*, *R. nipponica*, *R. kongmonthaensis*, *R. bulbicauda*, and *R. bermani*). The patterns of parasite–host switching and geographical distributions during the evolutionary history of *Rhabdias* ancestors is still an unsolved mystery. A more rigorous molecular phylogenetic study that includes broader representatives of *Rhabdias* species, especially these species collected from lizard hosts, is need to solve the above-mentioned issue.
